# Bruneian suicidal behaviors among school attending adolescents: a nationwide cross sectional study

**Published:** 2019-08

**Authors:** Nasrin Shahedifar, Masood A. Shaikh, Frederick Oporia, Michael Lowery Wilson

**Affiliations:** ^*a*^Injury Epidemiology and Prevention Research Group, Turku Brain Injury Center, Division of Clinical Neurosciences, Turku University Hospital and University of Turku, Turku, Finland.

## Abstract

**Background:**

It is aimed to determine the prevalence of suicidal behaviors regarding to epidemiological and psychosocial characteristics among Bruneian school-student adolescents.

**Methods:**

The cross-sectional study of 2599 (female: 49.9%; mean: 14.7 years old; SD: ±1.4) school-student adolescents aged between 13-17 years was based on the Global School-based Health Survey in Brunei. Data on suicidal behaviors, psychosocial and demographic characteristics were analyzed using multiple logistic regression model.

**Results:**

The prevalence of suicidal behaviors was 9.3%, 6.5% and 5.9% for suicidal ideation, suicidal plan and suicidal attempt respectively. Female adolescents reported more attempts (61.2%). It was found that some characteristics including suicide ideation (69%), anxiety (28%), loneliness (30%) were statistically different between the attempters of suicide and non-attempters among Bruneian school-student adolescents (p-value<0.001). Also, some suicide-related behaviors like planned suicide (52%), bullying victimization (21%), physical fight (29%), serious injury (28%), early sexual debut (8.5%), alcohol use at early age (20%), alcohol use in the past 30 days (12%) and physically attacked (30%) showed different rates between two groups of participants (p-value<0.001). Compared to those who did not report attempting suicide, the attempters were more likely to have suicide ideation (OR 10.58; 95% CI 5.10, 21.97); planned suicide (OR 9.82; 95% CI 4.60, 20.96); and sustained serious injury (OR 4.01; 95% CI 2.03, 7.93).

**Conclusions:**

The revealed suicidal behaviors, including being lonely, anxious, bullied, attacked physically, alcohol use among school students should be considered by parents and school and counselling service staff in order to prevent selectively.

**Keywords:**

Suicide, Adolescents, Brunei Darussalam

